# RD-Connect, NeurOmics and EURenOmics: collaborative European initiative for rare diseases

**DOI:** 10.1038/s41431-018-0115-5

**Published:** 2018-02-27

**Authors:** Hanns Lochmüller, Dorota M. Badowska, Rachel Thompson, Nine V. Knoers, Annemieke Aartsma-Rus, Ivo Gut, Libby Wood, Tina Harmuth, Andre Durudas, Holm Graessner, Franz Schaefer, Olaf Riess, RD-Connect consortium, NeurOmics consortium, EURenOmics consortium

**Affiliations:** 1 MRC Centre for Neuromuscular Diseases, Institute of Genetic Medicine, Newcastle University, Newcastle upon Tyne, UK; 2 Department of Neuropediatrics and Muscle Disorders, Medical Center–University of Freiburg, Faculty of Medicine, Freiburg, Germany; 3 Department of Genetics, Center for Molecular Medicine, University Medical Centre Utrecht, Utrecht, The Netherlands; 4 Leiden University Medical Center, Leiden, The Netherlands; 5 Centro Nacional de Análisis Genómico, Center for Genomic Regulation, CNAG-CRG, Barcelona Institute of Science and Technology, Barcelona, Spain; 6 Department of Medical Genetics and Applied Genomics, University of Tübingen, Tübingen, Germany; 7 CMAST bvba, Strategic Collaborations, Munich, Germany; 8 CMAST bvba, Strategic Collaborations, Temse, Belgium; 9 Centre for Rare Diseases, University of Tübingen, Tübingen, Germany; 10 Division of Pediatric Nephrology, Heidelberg University Center for Pediatrics and Adolescent Medicine, Heidelberg, Germany

## Abstract

Although individually uncommon, rare diseases (RDs) collectively affect 6–8% of the population. The unmet need of the rare disease community was recognized by the European Commission which in 2012 funded three flagship projects, RD-Connect, NeurOmics, and EURenOmics, to help move the field forward with the ambition of advancing -omics research and data sharing at their core in line with the goals of IRDiRC (International Rare Disease Research Consortium). NeurOmics and EURenOmics generate -omics data and improve diagnosis and therapy in rare renal and neurological diseases, with RD-Connect developing an infrastructure to facilitate the sharing, systematic integration and analysis of these data. Here, we summarize the achievements of these three projects, their impact on the RD community and their vision for the future. We also report from the Joint Outreach Day organized by the three projects on the 3rd of May 2017 in Berlin. The workshop stimulated an open, multi-stakeholder discussion on the challenges of the rare diseases, and highlighted the cross-project cooperation and the common goal: the use of innovative genomic technologies in rare disease research.

## Introduction

Although individually uncommon, rare diseases (RDs) collectively affect 6–8% of the population (ca. 30 million people in the European Union) [1, 2]. Medical interventions for RDs constitute a major part of healthcare spending [3]. Their rarity and diversity pose specific challenges for healthcare provision and research, and for the development and marketing of treatments. The unmet need of the rare disease community was recognized by the European Commission, who in 2012 funded three flagship projects, RD-Connect, NeurOmics and EURenOmics, to help move the field forward with the ideas of harmonization and data sharing at their core.

Genomics and other emerging omics technologies create potential for gene-based treatments and personalized medicine, which are particularly important for RDs, since 80% of RDs are genetic [2]. The capacity for genome and exome sequencing is growing rapidly and the limiting factor is now the ability to share and analyse these vast quantities of data rather than generate them. Despite the advances in computing technology, the processing and analysis of huge amounts of omics data remains challenging and requires new and innovative bioinformatics solutions and combined analysis of genetic and clinical data.

Harmonization and data sharing across research centers and across diseases is essential to advance knowledge, particularly for RDs, where patients are scarce and geographically disparate. Transnational and transdisease efforts are thus essential to make optimal use of resources. Patient registries, biobanks and bioinformatics analysis methods are the key infrastructure tools required for omics research. Hundreds of RD biobanks and patient registries already exist in Europe alone, and collaborative initiatives in specific disease groups have advanced the harmonization of these infrastructures several areas.

A continued bottleneck for cutting-edge RD-research, is that at present efforts of individual researchers continue to multiply while remaining largely “siloed”, with almost no information exchange. Genetic information, biomaterial availability, detailed clinical information (deep phenotyping) and research/trial datasets are hardly ever systematically connected beyond an individual research lab, and are rarely made accessible for reuse.

The International Rare Diseases Research Consortium (IRDiRC), launched in 2011, unites researchers and organizations investing in RD-research in two common goals: delivering 200 new therapies for RDs and the means to diagnose most RDs by the year 2020. The first goal has been achieved already in early 2017 [4]. However, some of these therapies target the same disease, while for the vast majority RD patients no treatment is available [4, 5]. Rarity remains the biggest bottleneck in therapy development for RDs.

Because of the large collective proportion of the population affected, and the significant burden they place on healthcare systems, RDs are a priority for research funded by the European Commission. Between 2007 and 2017, the EC funded €900 million in collaborative research projects on RDs including NeurOmics, EURenOmics, and RD-Connect [6]. NeurOmics and EURenOmics discovered jointly over 120 new disease genes, while RD-connect developed an infrastructure to facilitate the sharing and analysis of this data, which holds now data from almost 3000 individuals. This collaborative approach represents a new frontier of RD-research, diagnosis and therapy development. In this review, we present the accomplishments of these three projects, their role in the RD research and outlooks.

## RD-Connect

**Fig. 1:**
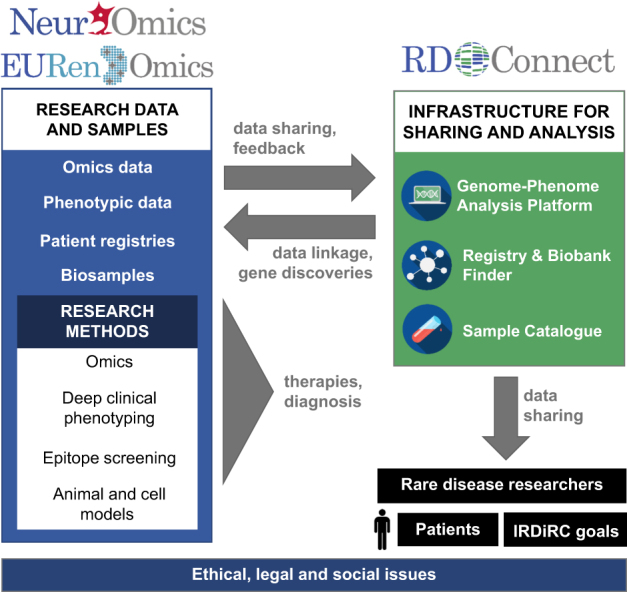
Collaboration between NeurOmics, EURenOmics and RD-Connect. Two research projects, NeurOmics and EURenOmics, generate large amounts of rare disease (RD) data (box “Research data and samples”) using a number of methods and approaches (box “Research methods”). These yield new therapies and improved diagnosis for RD patients. The omics and deep clinical phenotyping data are uploaded to the RD-Connect Genome-Phenome Analysis Platform (box “Infrastructure for sharing and analysis”), which allows their analysis leading and gene discovery. Researchers from the two projects also provide user feedback helpful in the Platform development. Information about patient registries and biobanks holding NeurOmics and EURenOmics patient data and biosamples are entered in the RD-Connect Registry & Biobank Finder, while detailed information about collected biosamples is shared via the RD-Connect Sample Catalogue. Those data are linked within the RD-Connect infrastructure are shared with the global RD research community, which facilitates rare disease research, diagnosis and therapy development and thus contributes to the IRDiRC goals. To address ethical, legal, and social issues, all three projects work together on developing appropriate best practices for international sharing of patient data

RD-Connect (www.rd-connect.eu) is a global research and infrastructure resource for RDs. Set up to overcome the siloing, fragmentation and inaccessibility of datasets from different rare disease projects, RD-Connect links omics data with phenotypic data and information in registries and biobanks at both an individual-patient and whole-cohort level. This enables researchers to analyse their own data and compare them with others to gain a complete view of their disease and patient population of interest. Data shared through RD-Connect are accessible beyond the usual institutional and national boundaries and researchers worldwide can benefit from the opportunity to work with others in the RD field, relate human phenotypes to a particular gene or pathway of interest, pool data to create larger cohorts, find confirmatory cases, and access samples for further study. This has been made possible through the strong collaboration with NeurOmics and EURenOmics (Fig. 1).

The integrated genome-phenome analysis platform developed by RD-Connect brings together anonymised omics and clinical data with tools and services to analyse this data online. The central portal provides access to the genomics analysis interface, the PhenoTips database [7] storing human phenotype ontology (HPO)-coded phenotypic profiles for individual cases [8]. It also provides a directory of biobanks and patient registries and a biosample catalogue that allows drill-down to details of individual samples hosted in participating biobanks.

### Genomics analysis interface

**Fig. 2:**
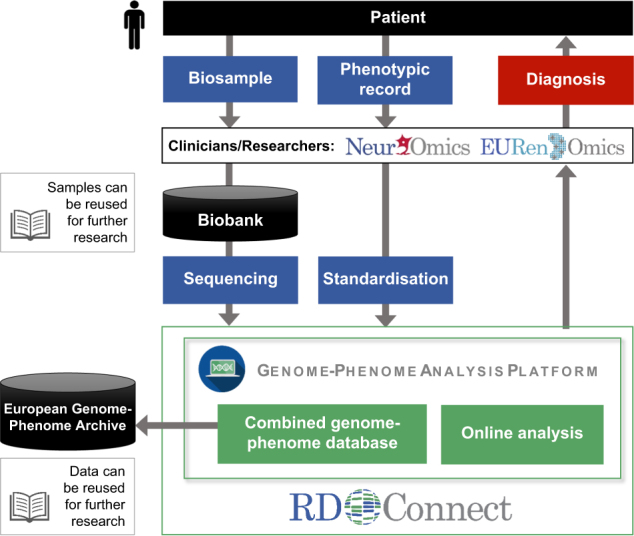
Data flow within the RD-Connect infrastructure. Samples donated by RD patients are deposited in biobanks, which allows their usage for further research. Sample sequencing produces omics data, which are uploaded to and processed in the RD-Connect Genome-Phenome Analysis Platform and deposited in the European Genome-Phenome Archive (EGA) for future reuse. In parallel, detailed patient phenotypic records are also uploaded to the platform and linked to the omics data. The platform allows clinicians and researchers to analyse combined genomic and phenotypic data to diagnose patients and discover novel disease genes and phenotypes

To let researchers reach the same results regardless of the sequencing provider used, RD-Connect re-processes raw data through a single pipeline to standardize variant calling and annotation. RD-Connect’s mechanism for sharing and analysis of RD genomic data begins with submission of the raw .bam or .fastq files, which is essential in order to allow data from multiple sequencing providers to be processed through the standard pipeline and to ensure comparability. The raw data are stored for long-term access at the European Genome-Phenome Archive (EGA) [9], a secure, controlled-access repository, while the processed data are made accessible online for real-time analysis in the RD-Connect genomics analysis interface. This workflow not only allows the researchers themselves to analyse their own cases but also ensures that the samples and data are accessible to others, thus maximizing the added value of the project for future research (Fig. 2).

Thanks to the collaboration with NeurOmics and EURenOmics, the Genome-Phenome Analysis Platform (https://platform.rd-connect.eu) is a rich resource containing whole exomes and genomes of a large number of individuals with rare neuromuscular (NMD) or neurodegenerative (NDD) (NeurOmics) and rare kidney disease (EURenOmics), but it is also growing rapidly for other RD groups, such as mitochondrial, neurogenetic and immunological disorders. In December 2017, the Platform contained exomes and genomes of almost 3000 cases, uploaded by numerous research projects, and the number is rapidly increasing.

RD-Connect believes that empowering disease experts to do their own analysis online will speed up diagnosis and gene discovery as well as give incentives for data sharing. This has proven a success as RD-Connect has already played a key role in the discovery of almost 20 published novel RD-genes and phenotypes. The Genome-Phenome Analysis Platform is a user-friendly tool for diagnosis and gene discovery, accessible to all, even those without experience in bioinformatics. A straightforward, secure registration process allows researchers to access the Platform to analyse and query their own data as well as data submitted by others. The data are accessible to authorized users following a predefined 6-month embargo period that gives researchers exclusive access to their own data before they are shared more widely.

A researcher can select one or multiple individuals (e.g., trios or other family relationships) to study and then filter and refine the results by mode of inheritance, population frequencies, in silico pathogenicity prediction tools, gene lists and ClinVar, HPO and OMIM codes. In addition, the integrated Exomiser tool extracts HPO terms describing the symptoms of the affected patients (from PhenoTips) and selects genes that match them.

The integrated matchmaker exchange system [10, 11] allows individuals with a variant in the gene of interest that may have been to comparing their symptoms and confirm a gene discovery. The Global Alliance for Genomics and Health’s “beacon” application programming interface (http://ga4gh.org/#/beacon) has been implemented, which enables querying of the presence or absence of single variants in the RD-Connect cohort.

The runs of homozygosity (RoH) feature allows identification of consanguineous cases even when not flagged as such by the treating clinician. To focus gene discovery search, RoH narrows the search down to only those genomic regions that are homozygous in the patient.

In addition to the resources offered through the Genome-Phenome Analysis Platform, RD-Connect partners have developed a number of bioinformatics tools to assist researchers in omics analysis and therapeutic target identification. These include variant analysis and annotation tools such as UMD-Predictor (http://umd-predictor.eu/) [12, 13], Human splicing finder (http://www.umd.be/HSF3/) [14], VarAFT (http://varaft.eu/) as well as therapeutic prediction tools and gene–drug interaction resources. The three tools have already contributed to over 1000 genetic studies. The genome–phenome analysis platform also contains the ALamut® Functional Annotations (ALFA) (Interactive Biosoftware, Rouen, France, http://www.interactive-biosoftware.com/)—a gene regulation prediction software tool designed to evaluate in silico potential effects of non-coding DNA variations.

### Biobanks and patient registries

RD-Connect activities in the patient registry arena focus on enabling data linkage across resources and on the principle of making registry data findable, accessible, interoperable and reusable (FAIR) [15]. This approach is gaining significant traction internationally through the European Open Science Cloud (www.eoscpilot.eu) and NIH Commons (https://commons.era.nih.gov/commons/). The rationale behind its application in the patient registry context is to allow computers to assist in analysis that otherwise is either impossible due to incompatibilities between datasets, or requires manual data aggregation and labor-intensive interrogation of the data.

To maximize the potential of patient registries and biobanks, RD-Connect has developed two complementary systems: the Registry & Biobank Finder (http://catalogue.rd-connect.eu/) [16] providing information about existing registries and biobanks worldwide, and the Sample Catalogue (https://samples.rd-connect.eu/) [17, 18] enabling searchable access to biosample records at an individual sample level. The RD-biobank network EuroBioBank [19] is the *de facto* biobank network for RD-Connect.

### Ethical, legal, and social issues and patient involvement

Sharing sensitive data for research reuse raises many ethical, legal, and social issues (ELSI). To address them, RD-Connect has developed ethical best practices that protect patient privacy without hindering research. ELSI experts within RD-Connect are engaged in establishing the ethical framework under which RD-Connect can enable sharing of sensitive human data in a secure and ethical fashion [20]. All researchers wishing to use the RD-Connect platform sign the RD-Connect code of practice based on legal requirements and ethical principles as well as patient and scientific needs.

Input of patient representatives into RD-Connect, NeurOmics, and EURenOmics activities is managed by EURORDIS through the Patient Advisory Council (PAC; http://rd-connect.eu/committee/pac/) and Patient and Ethics Council (PEC; http://rd-connect.eu/committee/rd-pec/), which have provided valuable guidance on the project’s direction, particularly in ethically challenging areas relating to data sharing where risk and benefit must be carefully evaluated. PAC members engage with each of the RD-Connect technical work packages, which not only enables the technical experts to have direct input from the PAC, but also strengthens the commitment and engagement of the PAC members, supports capacity building, and improves dissemination of the project’s outputs to the wider RD patient community.

RD-Connect along with NeurOmics and EURenOmics are embedded in European collaborative projects and work with research infrastructures, such as BBMRI [21] and Elixir (www.elixir-europe.org). The unique value of RD-Connect is that is has built RD community by integrating different stakeholders, such as scientists, clinicians, IT experts, ethicists, lawyers, and patients, who otherwise would have limited opportunities to work together. The success of RD-Connect makes it the flagship project of the European Commission.

## NeurOmics

NeurOmics (www.rd-neuromics.eu) investigates several rare neurodegenerative (NDD) and neuromuscular (NMD) diseases. It aims to improve the lives of patients by identifying new disease-causing genes, establishing new and standardized diagnosis tools, establishing biomarkers to monitor disease progression and treatment efficiency, finding new genetic modifiers of disease onset and course, and developing novel therapeutic approaches (Fig. 1).

The project has undertaken whole exome (WES) and whole genome sequencing (WGS) of 1105 samples, patients and family members, which resulted in the identification of over 100 new disease genes (Table 1), of which 89 are already published mostly in high ranking journals (Supplementary Table 2). For 43 genes, novel phenotypical associations were established in 50 independent publications (Supplementary Table 3). This achievement will greatly improve the diagnosis of rare neurodegenerative and neuromuscular diseases and will increase understanding of their pathogenesis.

**Table 1:** Research outputs of NeurOmics and EURenOmics

NeurOmics	
Samples analysed by WES/WGS	1105
Discovered disease genes^a^	>100
Novel phenotypical associations^b^	43
Diagnostic panels^c^	8
Genes covered in the panels	1663
Samples analysed by diagnostic panels	3840
Diseases covered by biomarker analysis	3
Therapeutic trials/studies (HD, MD, SCA, 2x HSP)	5
EURenOmics	
Families analysed by WES/WGS	315
Discovered disease genes and genomic rearrangements	37
Disease genes^d^	26
Genomic rearrangements	11
Diagnostic panels^e^	5
Genes covered in the panels	687
Samples analysed by diagnostic panels	>4000
Diseases covered by biomarker analysis	5
Therapeutic trials/studies	3

The research outputs of the two projects include novel disease genes, diagnostic panels, novel biomarkers, and therapeutic trials. Many of the gene discoveries were done with the contribution from RD-Connect

^a^ For the full list of the published novel disease genes published, see the Supplementary Table 2

^b^ See Supplementary Table 3

^c^ See Supplementary Table 4

^d^ For the full list of the published novel disease genes, see the Supplementary Table 5

^e^ See Supplementary Table 6

To understand the relationship between genes and the phenotype, NeurOmics researchers have entered data of 1550 patients into the PhenoTips system, which helps them to describe the clinical features using the standardized HPO terms. This allows matching patients with similar phenotypes and share the data through the PhenomeCentral database.

NeurOmics also combines -omics in animal models and cutting-edge cellular models (human-induced pluripotent stem cells) and uses mouse models in therapy development, e.g., antisense oligonucleotide-mediated exon skipping [22, 23].

To improve and speed up the sequencing of patients, NeurOmics has developed targeted NGS panels (Supplementary Table 4), which allow quick analysis of selected sets of all known genes associated with the disease of interest. The panels have already been used for diagnosis of over 700 patients. To monitor disease progression in patients, NeurOmics is investigating disease biomarkers and has developed profiling methods for metabolites and lipids in plasma and cerebrospinal fluid based on ultra-high performance liquid chromatography coupled high resolution mass spectrometry (UPLC-HRMS) [24, 25].

The great success of NeurOmics is a result of not only excellent science and best practice but also of strong collaboration and data sharing between partners from across the project.

**Table:** 

*Case study 1:* Data sharing helps to identify new genes associated with rare diseases.
Two patients suffering from proximal muscle weakness mainly in the lower limbs were analysed by two NeurOmics research teams in Newcastle and London. The first case, based in Newcastle, UK, was a man in his fifties, whose symptoms had been slowly progressing since childhood. The London case was a 4-year-old boy also presenting with weakness of the neck and spine. In both cases, muscle biopsy indicated congenital myopathy. Exome analysis revealed that both patients carried disease-causing variants in the gene *SRPK3*. Using a genome-phenome analysis platform for data sharing, the research teams discovered that they had patients with variants in the same gene and exchanged information. This strongly supported *SRPK3* as a disease-causing gene in these patients and will now allow personalized genetic counseling. Thanks to data sharing, researchers identified *SRPK3* as a congenital myopathy gene, which will help other researchers worldwide to diagnose similar patients in the future. [Töpf A et al. in preparation]

## EURenOmics

EURenOmics (www.eurenomics.eu) aims to improve the lives of patients living with rare kidney diseases through developing tools for more accurate diagnoses, prognosis and developing new and better therapies this population (Fig. 1).

EURenOmics performed WES in 315 families resulted in the discovery of 11 new genomic rearrangements and 26 novel disease genes (Table 1), of which 20 have been already published (Supplementary Table 5). The lower number of gene discoveries than in NeurOmics is attributed to lower genetic complexity of the kidney. In parallel, EURenOmics established several novel cellular and animal models, to study the biological mechanisms of rare kidney diseases. To improve diagnosis, EURenOmics has developed targeted NGS panels, covering 687 genes for rare kidney diseases (Table 1). Validated panels have been used in over 4000 cases and allowed diagnosis in 6–65% in various diseases (unpublished information, Supplementary Table 6).

Identification of the gene causing the patient’s disease is crucial for developing the right treatment or applying an already existing therapy for a different disease (see case study 2).

**Table:** 

*Case study 2:* Genetic diagnosis allows targeted therapies.
At the age of 6, a male patient was diagnosed with steroid-resistant nephrotic syndrome (SRNS)—a disease that causes abnormalities in kidney function and excess of protein in the urine (proteinuria), which often leads to kidney failure. Researchers in EURenOmics discovered that his symptoms are caused by variants in the gene *ADCK4*, which impair the biosynthesis of Coenzyme Q10 (CoQ10) and lead to a kidney-specific mitochondrial dysfunction. Supplementation with oral CoQ10 reduces proteinuria. The same variant was found in the patient’s sister, who also had proteinuria, but showed no symptoms. CoQ10 supplementation has been applied in both children to maintain the kidney function in long-term.
To facilitate genetic diagnosis, the researchers have developed NGS gene panels that allow doctors to quickly check whether a sick child has variants in genes known to be associated with certain kidney diseases. The screening of a cohort of 1000 children with SRNS revealed that up to 10% of the patients who receive a genetic diagnosis are affected by treatable hereditary defects of CoQ10 biosynthesis. Hence, a progressive kidney disease previously considered untreatable has turned into an easily treatable condition in at least some of the affected children, thanks to progress in gene discovery and routine genetic diagnostics [26].
The precise genetic diagnosis allowed for genetic counseling important for future family planning. The parents were recommended in-vitro fertilization with preimplantation diagnostics, which allowed them to get a healthy son.

The integration of patient databases and advances in gene diagnostics have allowed the collection of detailed information on the disease and patient symptoms (deep phenotyping) and genotype–phenotype association studies in the largest cohorts of genetically classified patients with various rare kidney diseases [27–36].

The consortium also increased the understanding of the pathophysiological role of common variants and therapeutic modification not only in kidneys but also in non-renal systemic and organ-limited diseases, e.g. the role of tubulopathy genes in hypertension, cardiovascular and stone disease [37]. Thus, rare disease research contributes to finding potential therapeutic targets also for common diseases.

Apart from multi-omics, EURenOmics is also focusing on epigenetic studies, which include leukocyte methylome, miRNome profiling and CHiP-Seq analysis of key binding sites to glomerular, tubular and developmental transcription factors and epigenetic modifiers.

**Table:** 

*Case study 3:* Developing best practice in consent.
The increasing use of new sequencing technologies and increased international data sharing create new opportunities for research and therapy development, but also require adjustments of the existing informed consent procedures. In RD-research, resources are scarce and it is critical to facilitate the access and reuse of the existing samples and data collections. In older collections, the consent forms did not consider the new genome sequencing technologies, the possibility of the return of incidental finding (e.g. detecting a variant that highly increases the risk of breast cancer) or research projects that involve commercial partners, such as the pharmaceutical industry. Without relevant consent, the samples and data cannot be used or reused for research. It is therefore essential to make efforts to re-contact patients, where required, to update their consent. The three projects have developed guidelines on the re-contacting procedure. Sometimes re-contacting is impossible, for example because the patient is deceased or where current contact details are not available. In such cases, the researchers should apply for permission to use the samples to institutional review boards/research ethics committees (IRBs/RECs) [20].

For prospective studies, the three projects created templates of consent documents, which should include key core elements and more broadly described research purposes with ongoing updates for participants. Investigators need to confirm that the consent for data sharing is in place when entering patients into databases.

Both EURenOmics and NeurOmics continue close collaboration with RD-Connect, particularly in data sharing, development of the HPO and standardization of ethical aspects, such as consent procedures.

## Conclusion

The establishment of the International Rare Diseases Research Consortium (IRDiRC) in 2011 was a milestone for the RD community. IRDiRC unites researchers and institutions investing in RDs research worldwide to improve patients’ lives by delivering new therapies for RDs and means to diagnose them. IRDiRC has developed “policies and guidelines” and recommends specific resources to enable and facilitate RD research [38, 39]. Since then, a significant progress has been made in research and diagnosis and, with respect to therapies, even more was achieved than initially expected. RD-Connect, NeurOmics and EURenOmics have greatly contributed to those successes by establishing new approaches in research, diagnosis and therapy development and an infrastructure that makes allows implementing them. However, despite of the great achievements of IRDiRC and contributing projects, most patients still lack diagnosis and treatment, and the RD community faces a number of challenges, which need to be addressed in near future [4].

## Electronic supplementary material

Report from the joint RD-Connect, NeurOmics and EURenOmics workshop (Outreach Day) on rare disease research

Novel genes published by the NeurOmics consortium

Genes with novel phenotypical associations identified by the NeurOmics consortium

Disease specific diagnostic gene panels developed and applied by NeurOmics

Novel Genes published by the EURenOmics consortium

Disease specific gene panels developed and applied by EURenOmics

RD-Connect Consortium list

NeurOmics Consortium list

EURenOmics Consortium list
