# Supplementary material for: RD-Connect, NeurOmics and EURenOmics: collaborative European initiative for rare diseases
Source: Eur J Hum Genet. 2018 Feb 27;26(6):778–85. doi: 10.1038/s41431-018-0115-5 (PMC5974013; doi:10.1038/s41431-018-0115-5)
Supplement: Supplementary file 1 — Report from the joint RD-Connect, NeurOmics and EURenOmics workshop (Outreach Day) on rare disease research [file 41431_2018_115_MOESM1_ESM.docx]

# Report from the joint RD-Connect, NeurOmics and EURenOmics workshop (Outreach Day) on rare disease research

## Introduction

To stimulate an open, multi-stakeholder discussion on the challenges of the rare diseases, the three EU flagship projects - RD-Connect, NeurOmics and EURenOmics – organised an Outreach Day. The workshop took place on the 3^rd^ of May 2017 in Berlin and highlighted the cross-project cooperation and the common goal: the use of innovative genomic technologies in rare disease research. The workshop brought together around 200 stakeholders from the EU, US, Australia and Japan, including researchers, clinicians, patient representatives, policy makers, members of research infrastructures and the pharmaceutical industry.

After the opening remarks from Iiro Eerola from the European Commission, the scientific project officer of the three projects, and the project coordinators, Olaf Riess, Franz Schaefer and Hanns Lochmüller, the program consisted of three panel sessions devoted to key objectives in rare disease research: data sharing, diagnostics and therapy. The panels of all sessions comprised the members of each of the three projects and patient representatives. The event finished with training sessions on the RD-Connect Genome-Phenome Analysis Platform and the use of the Sample Catalogue for biobanks.

## Data sharing

The first panel session, focusing on data sharing, was chaired by Ivo Gut, the director of Centro Nacional de Análisis Genómico in Barcelona, Spain, who is leading the work on the RD-Connect Genome-Phenome Analysis Platform. The panel consisted of Peter-Bram ‘t Hoen (Leiden University Medical Center, RD-Connect), Cathy Turner (Newcastle University, NeurOmics), Matthias Kretzler (University of Michigan, EURenOmics), Joseph Irwin (SMA Support UK, RD-Connect) and Veronica Popa (AHDS registry, Patient Advisory Council).

Ivo Gut opened the session stressing that data sharing across resources is essential to successful and meaningful research in RDs and involves numerous stakeholders: Patients and their families, Patient organisations, Funders, Clinicians, Researchers, Industry and others. Too often data is fragmented, siloed and inaccessible for research. Though there are still barriers to data sharing, many solutions now exist both in the tools available and the guidance around ethical, legal and social implications of sharing data.

The sharing of genomic and phenotypic data facilitates faster and more accurate diagnosis. The sharing of biosamples through biobanks advances therapy and biomarker development and the sharing of registry data accelerates inclusion of participants into clinical trials. In all cases, sharing data and samples helps to reduce cost and prevents duplication of effort. Furthermore, sharing enables engagement between experts and the wider community.

### Incentives for data sharing

Despite the obvious benefits, in the experience of the panel members many researchers are reluctant to share the data, particularly if they think it will affect their chances of publication or securing a competitive grant. Additional obstacles were discussed including the lack of appropriate infrastructure, time and effort needed to upload data to a research infrastructure, lack of interoperability and data standards and issues around privacy protection.

Peter-Bram ‘t Hoen suggested that incentives for data sharing could be created by scientific journals and funding agencies. GertJan van Ommen (Leiden University Medical Center) commented that some journals already stipulate deposit of data into open access databases, but the data are not easily accessible and therefore are not reused.

RD-Connect, NeurOmics and EURenOmics have all worked to reduce the barriers to data sharing and have developed and adopted a joint data sharing policy. Sequencing and clinical data are shared within the wider RD-research community via controlled access to the RD-Connect platform.

Peter-Bram ‘t Hoen suggested that another benefit of data sharing via research infrastructures is publishing negative data, most of which are never published due to journals’ preference for positive findings. Joseph Irwin contributed that a further benefit could be the sharing of placebo data from clinical trials, which could be valuable as high quality natural history data.

### Data quality

The session continued with a discussion around data quality and that control of this is an essential element of data sharing. Unedited information, such as false annotation of variants as pathogenic, may lead to misdiagnosis and have harmful consequences for the patient. However, researchers tend to give low priority to data quality standards in research infrastructures.^1^ To ensure high data quality, data need to be generated according to best practices and curated and benchmarked before entering databases. RD-Connect addresses this issue and ensures that data in the Genome-Phenome Analysis Platform meet high quality standards.^2^

### Data linkage

The session chair broadened the topic and raised the importance of linking various types of data from different sources. In the future, RD-Connect will link multiomics data stored at the European Bioinformatics Institute.^3^ Genomes and transcriptomes from the EGA, proteomes from PRIDE^4,5^ and lipidomes and metabolomes from MetaboLights^6–8^ will be connected to broader phenotype data on the level of the individual patient.

It was acknowledged that data linkage is extremely challenging, due to differences in data format and structure as well as differences in data sharing policies between countries. To address this, initiatives such as the Personal Locker system^9,10^ are being developed to allow patients to control the way information about their healthcare is stored and accessed. Clinicians and researchers will have access to non-sensitive data (such as metadata), but to access sensitive data they will need to request it the from the data owner, i.e. the patient. Upon the identification of the data user, the data owner will be able to decide which of the sensitive data to make accessible.

### Patient-centred data sharing and legal issues

Veronica Popa and Cathy Turner introduced the importance of patient input, and discussed how they can be the greatest advocates of RD-research. Many patients want their data to be used for research and benefit others, but they also want to be informed. Researchers should be obliged to be transparent about what happens with the patient data.

The panel agreed that many obstacles for data sharing are related to consent. RD-Connect and NeurOmics have done a lot of work ensuring that consents are appropriate for data sharing in platforms such as RD-Connect. When consent is given for a specific purpose, it can often prevent the reuse of the data in future studies, some of which may not be considered until many years later. This currently means that patients must be re-contacted if a new research need arises; however, this often resource intensive and inefficient. Future best practice should consider a broad consent covering the use of data in future research. The importance of data security was acknowledged; however, the panel discussed that overprotection of patient privacy could impede research and development of life-saving therapies.

The discussion highlighted some of RD-Connect’s work in this area. In particular, a study on the attitudes of rare disease patients to participation in genomics research, particularly around large-scale, international data and biosample sharing. The study concluded that patients see data sharing as imperative for the advancement of medical research.^11^

Furthermore, RD-Connect included experts in ethical and legal standards and together they have developed best ethical practices and recommendations for a regulatory framework for linking medical and personal data related to RD - in particular, the Code of Practice: an ethical framework to enable sharing of sensitive human data in a secure and ethical fashion, based on legal requirements and ethical principles as well as patient and scientific needs. The panel agreed these documents are important and should be considered widely to ensure rare disease research can have the greatest impact.

### New Challenges

The session concluded with a discussion around the changing environment of data sharing in rare diseases. The RD community will need to adapt to the new European General Data Protection Regulation (GDPR), which will enter into force on 25 May 2018.^12^ In response to the new legislation, the rare disease community including those involved in RD-Connect, NeurOmics and EURenOmics are working on a collaborative effort to produce a Code of Conduct for data sharing in biomedical research.

The launch of 24 European Reference Networks (ERNs) for rare diseases in March 2017 also presents new opportunities for data sharing. The tools and methodology applied in RD-Connect, NeurOmics and EURenOmics shows the benefits of harmonisation and collaborative working, RD-Connect is therefore well placed to provide a framework for genomic phenotypic registry data and support the ERNs in their research and diagnostic goals.

## Diagnosis

The diagnosis session was chaired by Nine Knoers (University Medical Centre Utrecht, EURenOmics). She was assisted by the panel members: Milan Macek (Charles University in Prague, RD-Connect), Peter Bauer (University of Tübingen, Centogene, NeurOmics) and Daniel Renault (FEDERG, Patient Advisory Council, Rare Disease Patient and Ethics Council).

Nine Knoers opened the session stating that genetic testing has an increasing role in diagnosing RDs and is crucial for genetic counselling, carrier testing, prenatal diagnosis, preimplantation genetic diagnosis (PGD) and identifying risk factors, and may be useful for determining appropriate treatment. The development of next generation sequencing (NGS) technologies (disease-specific multi-gene panels, WES and WGS) has made it possible to sequence multiple genes or even an entire genome in a single test. This has been followed by rapid development of tools for NGS data analysis, such as the RD-Connect Genome-Phenome Analysis Platform. This has greatly improved the likelihood and accuracy of diagnosis in RDs, but has also created novel challenges.

The introduction continued to discuss the burden of the diagnostic odyssey of RD patients. Diagnosis often takes years and most RD patients remain undiagnosed. IRDiRC aims to address this and create means to diagnose all RDs by 2020. However, as more disease genes are being discovered, the number of known RDs might exceed the expected 7000 RDs. That makes diagnosis a moving target.

### NGS data interpretation

The panel highlighted the complex nature of genetic diagnosis and that many of the challenges lie in the interpretation of the NGS data. Diagnosis is a multistep process that relies on finding a pathogenic genetic variant in an enormous amount of sequencing data. This requires adequate bioinformatic capacities and analysis tools. Furthermore, the discussion raised many other obstacles that increase the challenge of interpreting NGS data:

1. high numbers of Variants of Unknown Significance (VUS)
2. limited accuracy (less than 80%) of *In silico* tools for predicting variant pathogenicity (i.e. SIFT, Polyphen)
3. differing annotation in variant and variation databases^13^
4. relatively high frequency of designated pathogenic variants in healthy controls^14,15^
5. the purely qualitative nature of NGS, excluding any quantitative predictions

An audience member emphasized the importance of detailed clinical data (deep phenotyping) for the correct interpretation of omics data and annotation of pathogenic variants. An accurate phenotype can reveal the link between genetic variants and disease symptoms in addition to allowing the study of disease progression and comorbidities. The panel confirmed that this has been an important part of RD-Connect, NeurOmics and EURenOmics. The RD-Connect Genome-Phenome Analysis Platform is integrated with PhenoTips to collect detailed phenotypic information and with the Matchmaker Exchange, which enables the matchmaking of patients with similar mutations and phenotypes, leading to confirmation of pathogenicity of the identified variant. This also allows basic researchers with an interest in a particular gene to find human cases corresponding to their animal models in which the gene is affected. As many RDs are complex syndromes, analyses should also consider additional factors that may influence the disease course, such as the patient’s age and environment.

### Choosing the optimal diagnostic test

The choice of the optimal diagnostic test depends on the genetic complexity of the disease and the cost. The cost is particularly important, since in most countries diagnostic testing is not covered by the health insurance and the patients must pay for it themselves. Therefore, NeurOmics and EURenOmics have compared the different aspects of three diagnostic approaches: disease-specific multigene panels, WES and WGS (Tab 1). They showed that WGS, which is the most comprehensive method, has great diagnostic potential, but is also three times more expensive than WES.

| Test | Cost (Consumables) | Comments |
| --- | --- | --- |
| Panels | 250-350 € | High coverage. Advantage: no incidental findings |
| WES | ~ 800 € | Analysis of the coding sequence of all the genes in the genome, including some copy number variants. |
| WGS | ~ 1600 € | Analysis of the entire genome. Allows easy detection of copy number variants. Potentially important in research setting to discover clinically relevant variants that change gene expression. |

***Supplementary Table 1****. Comparison of three tests for genetic diagnosis. Whole Genome Sequencing (WGS) is the most efficient method, but is more expensive that Whole Exome Sequencing (WES).*

It is likely that WES (and possibly also WGS) will become the first-tier genetic test in due time because of the high overall diagnostic yield, especially in diagnostic odyssey cases, the relatively low costs, and the easy workflow (one test for all).^16,17^

Nevertheless, solving the unsolvable cases requires multi-omics approach, combining transcriptomics, epigenomics, metabolomics, phenomics etc. For instance, NeurOmics RNAseq studies revealed several possible genetic modifiers that may determine symptom severity and age of onset.

### NGS and privacy protection

As discussed in the earlier session data sharing is crucial to the advancement of care and therapy in rare diseases. This is also the case when looking at diagnostic data. In particular, when it comes to incidental findings (unanticipated, “secondary” findings not related to initial reason for genetic analysis) which are reported in 1-8.8% of cases in different studies Reporting them back to the patients can sometimes save their lives, but can also influence unaffected family members, who may share the same risk variants. The panel and audience acknowledged the complexity of this issue, however did not conclude on how to move forward. This is not an area that was explored in detail in RD-Connect, NeurOmics and EURenOmics, but is something for future consideration.

### Medical staff training and personalised genetic counselling

As stated by Daniel Renault, patients are searching not only for a quick diagnosis but also for information about the prognosis and disease management. Results should be shared with the patient carefully, and clinicians should be sensitive to how the patient responds to it. Our goal should be this kind of personalised counselling in all healthcare systems.

Nine Knoers pointed out that currently, insufficient numbers of clinicians and biologists are trained to interpret NGS data to enable them to help patients and families manage the disease and make informed reproductive choices. Therefore, it is urgently needed to develop guidelines for genetic counselling and training programs for clinicians, while NGS analysis should become a part of curriculum at all medical universities. Which professions are the most appropriate to provide genetic counselling remains under debate.

### Diagnosis across the globe

Matthias Kretzler raised the importance of considering genetic diagnosis in a global context. He stated that most genetic studies are currently performed in people of Caucasian ethnicity. We therefore lack data from other ethnic groups and this impedes the diagnosis of non-Caucasian patients. The bias is evident not only between continents, but also within the EU itself – as pointed by Milan Macek, patients from Eastern Europe are underrepresented in most studies. Several countries in Africa and Middle East have valuable high quality DNA and data collections and active RD communities, which makes them potential important research partners. Researchers and research funders, such as EU and NIH, are actively exploring these research opportunities across continents.

High costs of genetic testing create inequity in the access to diagnosis between patients from different parts of the world. In certain countries, genetic testing is covered by the health insurance, but in most, the patients have to cover the full cost or have no access at all.

## Therapies

The last panel session, dedicated to therapies, was chaired by Annemieke Aartsma-Rus (Leiden University Medical Center, NeurOmics). The panel was formed by Lucia Monaco (Fondazione Telethon, RD-Connect), Jack Wetzels (Radboud University Nijmegen Medical Centre, EURenOmics) and Jenny Versnel (Muscular Dystrophy UK, Patient Advisory Council).

Annemieke Aartsma-Rus opened the session with a reminder that the goal of IRDIRC to have 200 therapies for RDs by 2020 has been achieved ahead of time. However, some diseases have more than one therapy and with an estimated 6000-8000 RDs, it follows that for the vast majority of patients no therapy is yet available. Rarity remains the biggest bottleneck in therapy development for RDs. Therapy development for RDs is a complex and laborious process, because for most RDs tools required for clinical evaluation such as patient registries, trial site registries and outcome measures are not yet available. RD-Connect is currently addressing this by generating registry platforms. Still, many therapeutic options fail on the pre-clinical or clinical stage. We are beginning to look to new innovative approaches for therapy development and in some RDs these in a very early development stage, such as genome editing and induced pluripotent stem cells (iPSCs).

### Developing novel therapeutic approaches

One promising novel therapeutic approach highlighted by Annemieke Aartsma-Rus is antisense oligonucleotide (AON)-mediated exon skipping. This approach can be employed to correct the reading frame, to allow the production of a partially functional protein rather than a non-functional protein. This approach is in clinical trials for Duchenne muscular dystrophy and exon 51 skipping has been approved in the USA by the food and drug administration.^18^ For dysferlinopathies the approach can be used to bypass the mutation, while still allowing the production of an internally deleted dysferlin protein.^19^ This approach is being tested in vivo in a humanized dysterlin mouse model in the NeurOmics project. Finally, it is possible to use exon skipping to generate a less toxic protein, e.g. for spinocerebellar ataxia.^20^ This approach has been tested in vitro and in vivo in the NeurOmics project. The panel continued the discussion acknowledging it is not only the therapeutic approach itself that is required for therapy development but also the means to test is efficacy. The development of is essential to the advancement of individualized RD therapy**.** Biomarkers aim at accurate prediction of prognosis and treatment response. In EURenOmics, the levels of PLA2R antibodies in the blood of patients with membranous nephropathy were measured to predict spontaneous remission and allowed to avoid toxic, immunosuppressive therapy and use treatment with fewer side effects. Non-responders were identifiable by high levels of anti-PLA2R antibodies, while effective treatment was predicted by disappearance of PLA2R antibodies.^21^ Also, patients’ response to different kinds of treatment depended on the initial PLA2R antibody levels. This finding opens perspectives for antibody-guided, individualized therapy.

Lucia Monaco stated that biomarker and therapy development greatly benefit from biosample sharing. Cataloguing and registration of RD biobanks is an important part of the RD-Connect infrastructure. Sharing sample-level data in a common database, right down to individual samples, allows researchers to find the samples they need for their research and allows data from omics experiments to be traced back to the sample it came from for further research.

The panel discussed how therapy development can be further advanced through the use of the clinical bioinformatic tools that are integrated in the RD-Connect platform. They include: NR Analyzer (clinical bioinformatics system for the nonsense read-through therapeutic strategy), SKIP-e tool (to facilitate antisense oligonucleotide selection for exon skipping strategies), ePGA (electronic Pharmacogenomics Assistance system to provide information about gene-drug interactions).^22^

An additional approach to treatment for rare diseases is through drug repurposing. The NeurOmics coordinator, Olaf Horst Rieß, reported that NeurOmics has screened many compounds for therapy of selected diseases and in the future, the approach could be made more effective by screening multiple drugs for many diseases in a single test and sharing them between researchers.

### Tri-lateral communication between researchers, drug regulators and patients

Apart from the pharmaceutical industry, therapy development involves three major stakeholder groups: academics, regulators and patients. Efficient therapy development requires efficient tri-lateral communication between them, as each of has important information to contribute. Patients’ associations should communicate their needs with other stakeholders from the initial stages of therapy development and be involved in outcome measure and endpoint development and selection. Regulators, who approve drugs for all diseases, need to receive education on RDs and genetics, to understand the patients’ needs and novel therapy approaches. Multistakeholder meetings have recently been hosted by the European Medicines Agency for DMD and spinal muscular atrophy.^23,24^ Communication with patients at every step of the development process is crucial to facilitate therapy development, but also to let them have realistic expectations and consider acceptable and unacceptable burdens of therapy. Researchers, patients and regulators need to educate each other as is seen for example in the EURORDIS summer schools.

Therapy must be adjusted to patients’ needs. As pointed out by Annemieke Aartsma-Rus, patients’ priority is to increase their life quality rather than just life expectancy. Patients’ preference must be considered in drug development and clinical trials should have real-life outcome measures (i.e. ability to hold a cup rather than a 6-minutes walking test). Thus, patients must be involved from the earliest stage.

### Clinical trials

The session continued with a focus on clinical trials. Clinical trials depend on the capacity to recruit sufficient numbers of patients, which can be supported through patient registries that can provide access to large numbers of patients with a specific disease. To facilitate identification of registries holding information on a disease of interest, the RD-Connect ID-Cards catalogue provides information about existing registries and the numbers of patients they contain.

Annemieke Aartsma-Rus also mentioned the Care and Trial Site Registry (CTSR), originally developed as part of the Neuromuscular network TREAT-NMD and developed further by NeurOmics.^25,26^ The CTSR helps pharmaceutical companies and clinical researchers select trial sites and identify potential partners for upcoming research projects. To improve capacity for clinical trials, NeurOmics have expanded the CTSR to include potential trial sites for neurodegenerative conditions and introduced a “phenotype search facility”. The CTSR now has 337 sites in 51 countries and 88 of these see neurodegenerative disease patients. Around 67000 patients are seen across these 337 sites providing an invaluable resource to those conducting feasibility studies and seeking sites to carry out clinical trials.

### Treatment cost and availability

In response to a question from the audience the panel discussed the issue of cost in rare disease therapy development. The cost of therapy per patient is important and may cover one-off or lifetime treatment (e.g. gene therapy or enzyme replacement). It is expected that prices will decrease with advancements in technology. The RD community faces the challenge that even though the costs of developing treatment for rare and common diseases is similar, the per-patient prices of the RD medicines are much higher, because the recoup is spread between fewer patients.^27^ These costs are often unaffordable for the national healthcare systems and force the patients to find own resources. Researchers should therefore consider for potential therapies how expensive, invasive and likely to be translated to humans the therapy will be already at early stages of animal testing.

The access to therapy remains a challenge. While drugs for RDs are approved at an EU level, post marketing surveillance, marketing, pricing and health technology assessment (HTA) occurs at a national level, each with different requirements in different countries. Complicated procedures for HTA and a small size of the patient population might stop the company from applying for an approval in a given country and lead to inequalities. Therefore, EU should develop standards for drug marketing to ensure uniformity of access across countries. EURORDIS and the ERNs will be important players to address this challenge.

## Implementing research findings in cross-border healthcare

Olaf Rieß presented the potential future developments in the area of rare disease research. Research results and innovative therapy approaches need to be adapted in healthcare more efficiently. Thus, the EU plans to implement a research and innovation pipeline from bench to bedside. The Programme will link major EU and national initiatives: R&D, research infrastructures and patient registries, and bridge them to the ERNs to help implementing research results in healthcare.

The potential structure for the future Joint Programme Cofund initiative for European RD Research covers the following three key actions:

1. Transnational calls for proposals to fund collaborative RD-research projects, combining EC and Member States’ funding.
2. Data & Information – virtual platform for coordinated access, data exchange and repository facilities built on existing resources. Work on standards of reusability and regulatory readiness, analysis tools and links to care data as well as scalable pilots to ensure usefulness of the tools in clinical setting and ERNs.
3. Capacity building by training and support on data management, product development, translational research etc. for stakeholders including patient organisations. Sharing best practices and tech transfer facility towards industry.

NeurOmics and EURenOmics could become a blueprint for a “next generation” of global Research-Diagnostic-Clinical Networks, as their clinical partner organizations form the backbone of the ERNs ERN-EURO-NMD, ERN-RND and ERK-NET. The development such cross-border care system would increase equity in treatment accessibility across the EU.

**European Genetic Diagnostic Reference Networks** connecting centres to not only provide care but also diagnosis across borders. Like the ERNs, they would be connected via a Diagnostic Hub. RD-Connect plays a role in: 1) Establishing technical standards of WES/WGS in diagnostics; 2) Standardization of bioinformatic tools and algorithms; 3) Standards in reporting of findings; 4) Correction of wrong annotations in databases based on genetic diagnosis; 5) Harmonization of interpretations of variants; 6) Defining „actionable genes“ and harmonization for implementation into the clinics.

**European Research Core Networks** connecting laboratories, biobanks and other research centres coordinated by the European Joint Programme Brokerage hub.

The cooperation of such networks would allow research, diagnosis and care work in unison, e.g. coordinate preclinical studies in research units with biomarker studies and clinical trials. In parallel, EU member states should form diagnostic and care networks on the national level, which would provide patients in all parts of the country equal access to the expert centres.

## Conclusions

The Outreach Day provided an opportunity to multiple stakeholders, to exchange views and opinions on priorities, opportunities and challenges in the RD research field. It highlighted the importance of international RD-research and data sharing promoted by IRDiRC and funded by the European Commission, the NIH and other major funding organisations, with the ultimate goal of supporting and improving life quality of patients. RD-Connect, NeurOmics and EURenOmics have greatly contributed to three areas crucial in RD research: data sharing, diagnosis and new therapies. Whereas data was largely siloed prior to these projects, the collaboration and trust that has been built in the last years has culminated in clinical researchers benefitting from the computational systems established by RD-Connect that facilitate clinical research and might find their way into general clinical practice. For diagnosis of RDs, genetic testing has an increasing role and is likely to become the first step in diagnosing any RD. The growing use of next generation sequencing (NGS) techniques for genetic testing has increased the diagnostic efficiency, but has also created novel challenges, the most important being the interpretation of the enormous amount of NGS data being produced and novel ethical/legal issues. These challenges can only be addressed jointly, by data sharing and collaborative development of novel bioinformatic and analysis tools and by a joint multidisciplinary discussion on how to deal with the ethical/legal issues. One of the key challenges for RD therapy development is research using cell and animal models, while simultaneously establishing methods and tools to assess efficacy. Once drugs receive marketing authorization, a major challenge is to provide equal access to the therapy across different countries with different reimbursement systems. It is clear that both challenges can only be addressed by pan-European and worldwide multi-stakeholder collaboration. Finally, the novel approaches, established by the three projects, have laid foundations for the future developments in RD research, and may be adapted for research on common diseases.

# Works Cited

1 Battaglia S. ‘Medical Infrastructure / Users Forum’ (MIUF) Survey Report. 2017.http://www.ecrin.org/sites/default/files/CORBEL/MIUF%20Survey%20Lay%20Report.pdf.

2 Laurie S, Fernandez-Callejo M, Marco-Sola S *et al.* From Wet-Lab to Variations: Concordance and Speed of Bioinformatics Pipelines for Whole Genome and Whole Exome Sequencing. *Hum Mutat* 2016; **37**: 1263–1271.

3 Emmert DB, Stoehr PJ, Stoesser G, Cameron GN. The European Bioinformatics Institute (EBI) databases. *Nucleic Acids Res* 1994; **22**: 3445–3449.

4 Martens L, Hermjakob H, Jones P *et al.* PRIDE: The proteomics identifications database. *PROTEOMICS* 2005; **5**: 3537–3545.

5 Jones P. PRIDE: a public repository of protein and peptide identifications for the proteomics community. *Nucleic Acids Res* 2006; **34**: D659–D663.

6 Steinbeck C, Conesa P, Haug K *et al.* MetaboLights: towards a new COSMOS of metabolomics data management. *Metabolomics* 2012; **8**: 757–760.

7 Salek RM, Haug K, Conesa P *et al.* The MetaboLights repository: curation challenges in metabolomics. *Database* 2013; **2013**: bat029-bat029.

8 Haug K, Salek RM, Conesa P *et al.* MetaboLights—an open-access general-purpose repository for metabolomics studies and associated meta-data. *Nucleic Acids Res* 2013; **41**: D781–D786.

9 den Dunnen JT. The DNA Bank: High-Security Bank Accounts to Protect and Share Your Genetic Identity. *Hum Mutat* 2015; **36**: 657–659.

10 Wilkinson MD, Verborgh R, Bonino da Silva Santos LO *et al.* Interoperability and FAIRness through a novel combination of Web technologies. *PeerJ Comput Sci* 2017; **3**: e110.

11 McCormack P, Kole A, Gainotti S *et al.* ‘You should at least ask’. The expectations, hopes and fears of rare disease patients on large-scale data and biomaterial sharing for genomics research. *Eur J Hum Genet* 2016; **24**: 1403–1408.

12 Home Page of EU GDPR. EU GDPR Portal. http://eugdpr.org/eugdpr.org.html.

13 McCarthy DJ, Humburg P, Kanapin A *et al.* Choice of transcripts and software has a large effect on variant annotation. *Genome Med* 2014; **6**: 26.

14 Piton A, Redin C, Mandel J-L. XLID-Causing Mutations and Associated Genes Challenged in Light of Data From Large-Scale Human Exome Sequencing. *Am J Hum Genet* 2013; **93**: 368–383.

15 Nicolaou N, Pulit SL, Nijman IJ *et al.* Prioritization and burden analysis of rare variants in 208 candidate genes suggest they do not play a major role in CAKUT. *Kidney Int* 2016; **89**: 476–486.

16 Lionel AC, Costain G, Monfared N *et al.* Improved diagnostic yield compared with targeted gene sequencing panels suggests a role for whole-genome sequencing as a first-tier genetic test. *Genet Med* 2017. doi:10.1038/gim.2017.119.

17 Tan TY, Dillon OJ, Stark Z *et al.* Diagnostic Impact and Cost-effectiveness of Whole-Exome Sequencing for Ambulant Children With Suspected Monogenic Conditions. *JAMA Pediatr* 2017; **171**: 855–862.

18 Aartsma-Rus A, Krieg AM. FDA Approves Eteplirsen for Duchenne Muscular Dystrophy: The Next Chapter in the Eteplirsen Saga. *Nucleic Acid Ther* 2017; **27**: 1–3.

19 Barthélémy F, Blouin C, Wein N *et al.* Exon 32 Skipping of Dysferlin Rescues Membrane Repair in Patients’ Cells. *J Neuromuscul Dis* 2015; **2**: 281–290.

20 Toonen LJA, Schmidt I, Luijsterburg MS, van Attikum H, van Roon-Mom WMC. Antisense oligonucleotide-mediated exon skipping as a strategy to reduce proteolytic cleavage of ataxin-3. *Sci Rep* 2016; **6**. doi:10.1038/srep35200.

21 Dahan K, Debiec H, Plaisier E *et al.* Rituximab for Severe Membranous Nephropathy: A 6-Month Trial with Extended Follow-Up. *J Am Soc Nephrol* 2017; **28**: 348–358.

22 Lakiotaki K, Kartsaki E, Kanterakis A, Katsila T, Patrinos GP, Potamias G. ePGA: A Web-Based Information System for Translational Pharmacogenomics. *PLOS ONE* 2016; **11**: e0162801.

23 Straub V, Balabanov P, Bushby K *et al.* Stakeholder cooperation to overcome challenges in orphan medicine development: the example of Duchenne muscular dystrophy. *Lancet Neurol* 2016; **15**: 882–890.

24 Aartsma-Rus A, Balabanov P, Binetti L *et al.* Stakeholder collaboration for spinal muscular atrophy therapy development. *Lancet Neurol* 2017; **16**: 264.

25 Rodger S, Lochmüller H, Tassoni A *et al.* The TREAT-NMD care and trial site registry: an online registry to facilitate clinical research for neuromuscular diseases. *Orphanet J Rare Dis* 2013; **8**: 171.

26 Leary R, Oyewole A, Bushby K, Aartsma-Rus A. Translational Research in Europe for the Assessment and Treatment for Neuromuscular Disorders (TREAT-NMD). *Neuropediatrics* 2017; **48**: 211–220.

27 Medic G, Korchagina D, Young KE *et al.* Do payers value rarity? An analysis of the relationship between disease rarity and orphan drug prices in Europe. *J Mark Access Health Policy* 2017; **5**: 1299665.
