# Supplementary material for: RD-Connect, NeurOmics and EURenOmics: collaborative European initiative for rare diseases
Source: Eur J Hum Genet. 2018 Feb 27;26(6):778–85. doi: 10.1038/s41431-018-0115-5 (PMC5974013; doi:10.1038/s41431-018-0115-5)
Supplement: Supplementary file 2 — Novel genes published by the NeurOmics consortium [file 41431_2018_115_MOESM2_ESM.docx]

**Supplementary Table 2. Novel genes published by the NeurOmics consortium.** Over 100 novel disease genes have been discovered, of which 89 have already been published.

| Disease group | Gene | Publication |
| --- | --- | --- |
| Hereditary spastic paraplegia | *ATP13A2/PARK9* | Estrada-Cuzcano *et al*. Brain 2017 |
| Spinocerebellar ataxia | *DNAJC3* | Synofzik *et al*. AJHG 2014 |
| Spinocerebellar ataxia | *KCNA2* | Syrbe S *et al*. Nat Genet 2015 |
|  |  | Helbig *et al*. Ann Neurol 2016 |
| Spinocerebellar ataxia | *PNPLA6* | Synofzik *et al*. Brain 2014 |
| Spinocerebellar ataxia | *WWOX* | Mallaret *et al*. Brain 2014 |
| Ataxia | *POLR3A* | Minnerop *et al*. Brain 2017 |
| Hereditary spastic paraplegia | *B4GALNT1* | Boukhris *et al*. AJHG 2013 |
| Spinocerebellar ataxia/Hereditary spastic paraplegia | *GBA2* | Martin *et al*. AJHG 2013 |
| Facioscapulohumeral muscular dystrophy | *DNMT3B* | van den Boogaard *et al*. Am J Hum Genet 2016 |
| Limb-girdle muscular dystrophy/CMD | *MICU1* | Logan *et al*. Nat Genet 2013 |
| Facioscapulohumeral muscular dystrophy | *SMCHD1* | Lemmers *et al*. Nat Genet 2012, |
|  |  | Sacconi *et al*. AJHG 2013, |
|  |  | Lemmers *et al*. Hum Mutat 2015 |
| Spinal muscular atrophy/Lower motor neuron disease | *PIEZO2* | Delle Vedove *et al*. Am J Hum Genet 2016 |
| Congenital myasthenic syndrome | *VAMP1* | Salpietro *et al*. Ann Neurol 2017 |
| Autosomal recessive cerebellar ataxias | *CHP1* | Mendoza Ferreira *et al*. Neurol Genet 2017 |
| Spinal muscular atrophy and lower motor neuron disease | *BICD2* | Neveling *et al*. AJHG 2013, |
|  |  | Peeters *et al*. AJHG 2013, |
|  |  | Novarino *et al*. Science 2014 |
| Bethlem myopathy | *COL12A1* | Hicks *et al*. HMG 2013 |
| Muscular dystrophies | *INPP5K* | Wiessner *et al*. AJHG 2017 |
| Congenital myasthenic syndrome | *MYO9A* | O’Connor *et al*. Brain 2016 |
| Congenital myasthenic syndrome | *SLC25A1* | Chaouch *et al*. J Neuromusc Dis 2014 |
| Congenital myasthenic syndrome | *SLC5A7* | Bauche *et al*. AJHG 2016 |
| Congenital myasthenic syndrome | *SYT2* | Herrmann *et al*. AJHG 2014 |
| Congenital myopathies | *ZAK1* | Vasli *et al*. Brain 2016 |
| Congenital muscular dystrophies | *B3GALNT2* | Stevens *et al*. AJHG 2013 |
| Congenital myopathies | *CACNA1S* | Schartner *et al*. Acta Neuropathol. 2017 |
| Congenital muscular dystrophies | *GMPPB* | Carss *et al*. AJHG 2013 |
| Limb-girdle muscular dystrophy | *ISPD* | Cirak *et al*. Brain 2013 |
| Congenital muscular dystrophies | *SGK196* | Yoshida-Moriguchi *et al*. Science 2013 |
| Neuropathy | *SIL1* | Byrne *et al*. Neuromuscul Disord 2015 |
| BVVL/Riboflavin responsive | *SLC52A1-A3* | Manole *et al*. Brain 2017 |
| Hereditary spastic paraplegia | *SPG11* | Manole *et al*. J Neurol 2016 |
| Myopathy and ataxia | *MSTO1* | NaSpinocerebellar ataxia *et al*. Hum Mutat. 2017 |
| CMY | *SCN4A* | Zaharieva *et al*. Brain 2016 |
| Ataxia | *CAPN1* | Wang *et al*. Cell Rep 2016 |
| Spinocerebellar ataxia | 7.5-Mb duplication at chromosome 11q21-11q22.3 | Johnson *et al*. Mov Disord 2015 |
| Hereditary spastic paraplegia | *ALDH18A1* | Coutelier *et al*. Brain 2015 |
| Hereditary spastic paraplegia | *C19ORF12* | Landoure *et al*. Hum Mutat 2013 |
| Spinocerebellar ataxia | *CACNA1G* | Coutelier *et al*. AJHG 2015 |
| Hereditary spastic paraplegia | *CPT1C* | Rinaldi *et al*. JAMA Neurol 2015 |
| Spinocerebellar ataxia | *ELOVL5* | Di Gregorio *et al*. AJHG 2014 |
| Spinocerebellar ataxia | *GRID2* | Coutelier *et al*. Neurology 2015 |
| Hereditary spastic paraplegia | *IBA57* | Lossos *et al*. Neurology 2015 |
| Huntington's disease spectrum | *JPH3, CACNA1A, VPS13A, UBQLN2, VCP* | Mariani *et al*. JAMA Neurol 2016 |
| Hereditary spastic paraplegia | *KIF1C* | Novarino *et al*. Science 2014, |
|  |  | Dor *et al*. J Med Genet 2014, Caballero Oteyza *et al*. Neurology 2014 |
| Hereditary spastic paraplegia | *MAG, NT5C2, DDHD2, USP8, WDR48, ARL6IP1, ERLIN1, AMPD2, ENTPD1, ARSI, PGAP1, FLRT1, RAB3GAP2, MARS, ZFR1* | Novarino *et al*. Science 2014 |
| Hereditary spastic paraplegia | *PTK-THOC gene fusion* | Di Gregorio *et al*. J Med Gen 2013 |
| Hereditary spastic paraplegia | *REEP2* | Esteves *et al*. AJHG 2014; |
|  |  | Novarino *et al*. Science 2014 |
| Spinocerebellar ataxia | *TMEM240* | Delplanque *et al*. Brain 2014 |
| Dystonia, cerebellar atrophy, cardiomyopathy | *TOR1AIP1* | Dorboz *et al*. Orphanet J Rare Dis 2014 |
| Hereditary motor neuropathy/Charcot-Marie-Tooth disease | *ATL3* | Kornak *et al*. Brain 2014 |
| Hereditary motor neuropathy | *HARS* | Brozkova *et al*. Brain 2015 |
| Hereditary motor neuropathy/Charcot-Marie-Tooth disease | *SCN11A* | Leipold *et al*. Nat Genet 2013 |
| Hereditary motor neuropathy/Charcot-Marie-Tooth disease | *WARS* | Tsai *et al*. Brain 2017 |
| Spinocerebellar ataxia | *SYNE1* | Mademan *et al*. Brain 2016 |
| Muscular dystrophy | *POPDC1* | Schindler *et al*. J Clin Invest 2016 |
| Arthrogryposis | *ADGRG6* | Ravenscroft *et al*. AJHG 2015 |
| Myopathy | *CYC1* | Gaignard *et al*. AJHG 2013 |
| Myopathy | *KLHL40* | Ravenscroft *et al*. AJHG 2013 |
| Myopathy | *KLHL41* | Gupta *et al*. AJHG 2013 |
| Myopathy | *LMOD3* | Yuen *et al*. J Clin Invest. 2014 |
| Myopathy | *MTOR* | Baynam *et al*. AJMG 2015 |
| Myopathy | *PPA2* | Guimier *et al*. AJHG 2016 |
| Myopathy | *PYROXD1* | O'Grady *et al*. AJHG 2016 |
| Myopathy | *SPEG* | Agrawal *et al*. AJHG 2014 |
