# Supplementary material for: RD-Connect, NeurOmics and EURenOmics: collaborative European initiative for rare diseases
Source: Eur J Hum Genet. 2018 Feb 27;26(6):778–85. doi: 10.1038/s41431-018-0115-5 (PMC5974013; doi:10.1038/s41431-018-0115-5)
Supplement: Supplementary file 3 — Genes with novel phenotypical associations identified by the NeurOmics consortium [file 41431_2018_115_MOESM3_ESM.docx]

**Supplementary Table 3. Genes with novel phenotypical associations identified by the NeurOmics consortium.**

| Disease group | Phenotype | Gene | Publication |
| --- | --- | --- | --- |
| Spinal muscular atrophy and lower motor neuron disease | SMALED2 | *BICD2* | Synofzik et al. JNNP 2013 |
| Myopathy | Myopathy with slow progression | *BICD2* | Unger et al. Neurology 2016 |
| Spinal muscular atrophy and lower motor neuron disease | Lethal arthrogryposis, Asymptomatic | *BICD2* | Storbeck et al. EJHG 2017 |
| Lower motor neuron disease | HMSN | *MCM3AP* | Karakaya et al. Brain 2017 |
| Myopathy | MPD1 | *MYH7* | Komlosi et al. J Neuromuscul Dis 2014 |
| Spinal muscular atrophy and lower motor neuron disease | Neurodegeneration and secondary microcephaly | *PRUNE1* | Karakaya et al. Brain 2017 |
| Congenital myasthenic syndrome | Congenital myasthenic syndrome | *ANO10* | Balreira et al. J Neurol 2014 |
| Muscular dystrophies | Myopathy | *MEGF10* | Harris et al. Neuromuscul Disord. 2017 |
| Myopathy | Myopathy, tubular aggregate; Stormorken syndrome | *STIM1* | Harris et al. Neuromuscular disorder 2017 |
| Leukoencephalopathy |  | *AARS2* | Lynch et al. JAMA Neurol 2016 |
| HMN/CMT | CMT2S | *IGHMBP2* | Cottenie et al. AJHG 2014 |
| Hereditary spastic paraplegias | SPG9 | *ALDH18A1* | Coutelier et al. Brain 2015 |
| Autosomal recessive cerebellar ataxias | Recessive ataxia | *POLG2* | Van Marldergem et al. Ann Clin Translational Neurol 2016 |
| Autosomal recessive cerebellar ataxias | Recessive ataxia | *SLC25A46* | Hammer et al. Neurodeg Dis 2017 |
| Hereditary spastic paraplegias |  | *TFG/SPG57* | Elsayed et al. Eur J Hum Genet. 2016 |
| Spinal muscular atrophy and lower motor neuron disease | SMALED1 | *DYNC1H1* | Peeters et al. Hum Mutat 2015 |
| Charcot-Marie-Tooth disease type 2F/ Hereditary motor neuronopathies | HMN | *HSPB1* | Echaniz-Laguna et al. Human Mutation 2017 |
| Amyotrophic lateral sclerosis | HMN | *HSPB1* | Capponi et al. Human Mutation 2016 |
| Charcot-Marie-Tooth disease type 2F/ Hereditary motor neuronopathies | HMN | *HSPB8* | Echaniz-Laguna et al. Human Mutation 2017 |
| Charcot-Marie-Tooth disease type 2G | HMSN | *LRSAM1* | Peeters et al. Annals of Neurology 2016 |
| Hereditary motor neuronopathies/ Spinocerebellar Ataxias | HSN1E, ADCADN | *DNMT1* | Baets et al. Brain 2015 |
| Neuropathy | AMC with polymicrogyria | *BICD2* | Ravenscroft et al. Neuromuscul Disord. 2016 |
| Myopathy | Myalgia & rhabdomyolysis | *CAV3* | Scalco et al. Neuromuscul Disord 2016 |
| Muscular dystrophies | MDCMC | *CHKB* | Cabrera-Serrano et al. Muscle Nerve 2015 |
| Myopathy | Distal myopathy +Cystinosis | *CTNS* | Cabrera-Serrano et al. Neuromuscul Disord. 2017 |
| Myopathy | Congenital Myopathy | *DYNC1H1* | Beecroft et al. Neuromuscul Disord. 2017 |
| Distal arthrogryposis | DA5D | *ECEL1* | Barnett et al. Am J Med Genet 2014 |
| Myopathy | Limb-girdle muscular dystrophy | *GMPPB* | Cabrera-Serrano et al. Brain 2015 |
| Myopathy | Distal & Proximal myopathy with ophthalmolegia | *MYH2* | Cabrera-Serrano et al. Clin Genet 2015 |
| Myopathy | CMD1S | *MYH7* | Finsterer et al. Int J Cardiol 2014 |
| Myopathy | MPD1 | *MYH7* | Lamont et al. Hum Mutat 2014 |
| Fetal akinesia deformation sequence | Lethal multipl pterygium syndrome | *NEB* | Abdalla et al. Neuromuscul Disord. 2017 |
| Myopathy | DA1, DA2B, NEM4 | *TPM2* | Marttila et al. Hum Mutat 2014 |
| Myopathy | NEM1, CFTD | *TPM3* | Marttila et al. Hum Mutat 2014 |
| Myopathy | Hypercontractile congenital myopathy | *TPM3* | Donkervoort et al. Ann Neurol 2015 |
