# Supplementary material for: RD-Connect, NeurOmics and EURenOmics: collaborative European initiative for rare diseases
Source: Eur J Hum Genet. 2018 Feb 27;26(6):778–85. doi: 10.1038/s41431-018-0115-5 (PMC5974013; doi:10.1038/s41431-018-0115-5)
Supplement: Supplementary file 4 — Disease specific diagnostic gene panels developed and applied by NeurOmics [file 41431_2018_115_MOESM4_ESM.docx]

**Supplementary Table 4. Disease specific diagnostic gene panels developed and applied by NeurOmics.** The table presents the numbers of the genes covered in the latest versions of the panels (December 2017). Several diagnostic panels were developed in parallel by different groups within the consortium, and cover several overlapping genes, for example two Ataxia panels were created by the groups in Tübingen and Paris and two more general panels, designed by the groups in Marseille and Perth, covered ataxia genes among other genes.

| Diagnostic panel | Number of genes covered by panel | Number of samples | Success rate |
| --- | --- | --- | --- |
| Lower motor neuron disease (Cologne) | 65 | 35 | 17% |
| Hereditary spastic paraplegia (Tübingen) | 192 | 68 | 37% |
| Hereditary spastic paraplegia (Paris) | 74 | 774 | 30% |
| Ataxia (Tübingen) | 201 | 107 | 19% |
| Ataxia (Paris) | 34 | 412 | 14% |
| Neuromuscular disease (Cologne) | 475 | 62 | 45% |
| Neuromuscular disease (Marseille) | 306 | 112 | 33% |
| PathWest targeted panel for muscle and nerve (Perth) | 316 | 2270 | 32% |
