# Supplementary material for: RD-Connect, NeurOmics and EURenOmics: collaborative European initiative for rare diseases
Source: Eur J Hum Genet. 2018 Feb 27;26(6):778–85. doi: 10.1038/s41431-018-0115-5 (PMC5974013; doi:10.1038/s41431-018-0115-5)
Supplement: Supplementary file 5 — Novel Genes published by the EURenOmics consortium [file 41431_2018_115_MOESM5_ESM.docx]

**Supplementary Table 5. Novel Genes published by the EURenOmics consortium.** *in collaboration with group Lifton, New Haven, USA. **in collaboration with group Hildebrandt, Boston, USA. ***in collaboration with group Konrad, Munster, Germany.

| Disease group | Phenotype | Gene | Publication |
| --- | --- | --- | --- |
| Complement disorders | atypical hemolytic uremic syndrome (novel phenotype associated to *DGKE*) | *DGKE* | Azukaitis et al., J Am Soc Nephrol 2017* |
| Complement disorders | atypical hemolytic uremic syndrome (novel phenotype associated to *INF2*) | *INF2* | Challis et al., J Am Soc Nephrol 2017 |
| Congenital Anomalies of the Kidney and Urinary Tract (CAKUT) | Bilateral kidney agenesis | *ITG8A* | Humbert et al., Am J Hum Genet 2014 |
| Congenital Anomalies of the Kidney and Urinary Tract (CAKUT) | Bilateral kidney agenesis, uterus agenesis | *GREB1L* | De Tomasi, Am J Hum Genet, in press |
| Congenital Anomalies of the Kidney and Urinary Tract (CAKUT) | Kidney hypoplasia, deafness, developmental delay, dysmorphism | *PBX1* | Heidet et al., J Am Soc Nephrol 2017 |
| Nephrotic syndrome | Galloway-Mowat Syndrome | *LAGE3* | Braun et al., Nat Genet 2017** |
| Nephrotic syndrome | Galloway-Mowat Syndrome | *OSGEP* | Braun et al., Nat Genet 2017** |
| Nephrotic syndrome | Galloway-Mowat Syndrome | *TP53RK* | Braun et al., Nat Genet 2017** |
| Nephrotic syndrome | Galloway-Mowat Syndrome | *TRPKB* | Braun et al., Nat Genet 2017** |
| Nephrotic syndrome | Galloway-Mowat Syndrome | *WDR73* | Colin et al., Am J Hum Genet 2014 |
| Nephrotic syndrome | Steroid resistant nephrotic syndrome / Focal Segmental Glomerulosclerosis | *ADCK4* | Ashraf et al., J Clin Invest 2013** |
| Nephrotic syndrome | Steroid resistant nephrotic syndrome / Focal Segmental Glomerulosclerosis | NUP205 | Braun et al., Nat Genet 2016** |
| Nephrotic syndrome | Steroid resistant nephrotic syndrome / Focal Segmental Glomerulosclerosis | NUP93 | Braun et al., Nat Genet 2016** |
| Nephrotic syndrome | Steroid resistant nephrotic syndrome / Focal Segmental Glomerulosclerosis | *TTC21B* | Huynh Cong et al., J Am Soc Nephrol 2014 |
| Nephrotic syndrome | Steroid resistant nephrotic syndrome / Focal Segmental Glomerulosclerosis | XPO5 | Braun et al., Nat Genet 2016** |
| Nephrotic syndrome | Steroid resistant nephrotic syndrome, ichtyosis, adrenal insufficiency | *SGPL1* | Lovric et al., J Clin Invest 2017** |
| Renal tubular disorders | HELIX syndrome | *CLDN10B* | Hadj-Rabia et al., Genet Med 2017 |
| Renal tubular disorders | Idiopathic infantile hypercalcemia | *SLC34A1* | Schlingmann et al., J Am Soc Nephrol 2016*** |
| Renal tubular disorders | Polycystic Kidney Disease with hyperinsulinemic hypoglycemia | *PMM2* (promotor) | Cabezas et al., J Am Soc Nephrol 2017 |
| Renal tubular disorders | Renal Fanconi syndrome | *EHHADH* | Klootwijk et al., N Engl J Med 2014 |
