# Supplementary material for: RD-Connect, NeurOmics and EURenOmics: collaborative European initiative for rare diseases
Source: Eur J Hum Genet. 2018 Feb 27;26(6):778–85. doi: 10.1038/s41431-018-0115-5 (PMC5974013; doi:10.1038/s41431-018-0115-5)
Supplement: Supplementary file 6 — Disease specific gene panels developed and applied by EURenOmics [file 41431_2018_115_MOESM6_ESM.docx]

**Supplementary Table 6. Disease specific gene panels developed and applied by EURenOmics.** For Congenital abnormalities of the kidney and urinary tract (CAKUT), two panels were developed by and University Medical Center Utrecht (panel I) and by INSERM (panel II).

| Disease panel | Number of genes covered by panel | Number of samples | Success rate |
| --- | --- | --- | --- |
| Steroid resistant nephrotic syndrome | 34 | >1700 | 21% |
| Renal tubular disorders | 44 | >600 | 65% |
| Complement disorders | 13 | >750 | 40% |
| Congenital abnormalities of the kidney and urinary tract (CAKUT) – panel I | 208 | >900 | 6% |
| Congenital abnormalities of the kidney and urinary tract (CAKUT) – panel II | 388 | >300 | 18% |
